# Knowledge, Attitude, and Practices Towards Hepatitis B Infection Among Nursing Students: A Cross‐Sectional Study in Jordan

**DOI:** 10.1002/hsr2.71967

**Published:** 2026-03-04

**Authors:** Nader Alaridah, Raba'a F. Jarrar, Rayan M. Joudeh, Mallak Aljarawen, Hasan Nassr, Rahaf A. Jereisat, Arwa battah, Mohammad Jum'ah, Noor Rajeh Abu Hantash, Mohammad Nour Amr, Haneen Al‐Abdallat, Layan Ismail, Anas Y. El‐Massad, Heba Mahmmoud, Anas H. A. Abu‐Humaidan

**Affiliations:** ^1^ Department of Pathology, Microbiology, and Forensic Medicine, School of Medicine University of Jordan Amman Jordan; ^2^ Department of Clinical Laboratory Sciences, School of Science University of Jordan Amman Jordan; ^3^ Fellow in Ibn Sina University for Medical Sciences Amman Jordan; ^4^ School of Medicine The University of Jordan Amman Jordan; ^5^ College of Medicine Sulaiman Al‐Rajhi University Al‐Bukayriah Al‐Qassim Saudi Arabia; ^6^ HCA Houston Healthcare Clearlake University of Houston Houston USA; ^7^ Department of Medical Education, New York Medical College Saint Michael's Medical Center Newark New Jersey USA; ^8^ Internal Medicine Department Istishari Hospital Amman Jordan; ^9^ School of Medicine V.N. Karazin National University Kharkiv Ukraine; ^10^ School of Pharmacy Jordanian University of Science and Technology Irbid Jordan

**Keywords:** attitude, hepatitis B virus, Jordan, knowledge, nursing students, practices

## Abstract

**Background and Aims:**

Hepatitis B is a serious, communicable liver disease resulting from hepatitis B virus infection. Healthcare workers (HCWs), including nursing students, are at elevated risk of exposure. We assessed Jordanian nursing students' knowledge, attitudes, and practices (KAP) toward HBV and explored predictors of better KAP.

**Methods:**

We conducted a cross‐sectional online survey (March–August 2022) among 617 nursing students (years 3–5) at two Jordanian universities using a previously validated questionnaire (43 knowledge, 8 attitude, 3 practice items). Scores ≥ 70% were classified as “good.” Descriptive statistics and χ² tests were used, and multivariable logistic regression examined associations with KAP (α = 0.05).

**Results:**

Overall knowledge was satisfactory, particularly for transmission routes; 73.1% answered diagnostic items correctly. Misconceptions persisted about oral and airborne transmission (≤ 50% correct). Only 8.1% and 18.6% correctly identified treatment criteria and the urgency of treatment, respectively. Most students reported prior HBV vaccination (75.7%) and personal protective equipment use during patient contact (~71%); however, only 45.4% had undergone anti‐HBV testing before clinical rotations. In multivariable analyses, higher academic year, prior HBV‐related coursework, and clinical encounters with HBV patients were associated with better KAP (all *p* < 0.05).

**Conclusion:**

Jordanian nursing students demonstrated acceptable knowledge of HBV transmission but notable gaps in treatment knowledge, newborn immunization timing, and safe sharps disposal. Curricular enhancements should correct misconceptions about non‐transmission via food or air, reinforce post‐vaccination antibody testing and revaccination of nonresponders, and strengthen training on treatment indications and neonatal prophylaxis. Targeted education—particularly in earlier academic years—may improve HBV‐related KAP among future HCWs.

**Abbreviations**
CHB: chronic hepatitis B
dsDNA: double‐stranded deoxyribonucleic acid
HBV: hepatitis B viruses
HCWs: healthcare workers
IRB: Institutional Review Boards
KAP: knowledge, attitude and practices
SPSS: Statistical Package for the Social Sciences
S.D: standard deviation
WHO: World Health Organization

## 1 Introduction

Viral hepatitis B stands as an infectious liver disease caused by the double‐stranded deoxyribonucleic acid (dsDNA) hepatitis B virus (HBV) [1]. According to the World Health Organization (WHO), an estimated 296 million people were living with chronic hepatitis B infection globally in 2019, resulting in over 820,000 deaths each year [2].

The disease spreads through various means such as unsterilized surgical instruments, needle stick injuries, transfusion of infected blood and plasma, unprotected sexual intercourse, and vertical transmission from an infected mother during birth [3]. Although treatments for HBV exist, they are not curative and often necessitate long‐term management [4]. Consequently, the WHO has targeted the elimination of viral hepatitis, including HBV infection, by 2030 [5]. Knowledge entails understanding its etiology, mode of transmission, signs, symptoms, diagnosis, treatment, complications, and the importance of vaccination [6]. Attitudes toward prevention are shaped by perceived risk, threats, and the severity of the infection [7]. Practical preventive measures involve activities such as screening, vaccination, testing for antibodies after vaccination, avoiding blood exposure, preventing needle stick injuries, and ensuring proper glove change between patients [8]. Approximately three million HCWs globally face this risk annually, resulting in around 70,000 HBV infections [9]. Notably, HCWs, including nursing students, face a fourfold greater risk of HBV infection compared with non‐exposed individuals, often due to the lack of experience, inadequate training, and lapses in adherence to safety protocols [10]. Previous studies indicated a high level of awareness among HCWs regarding the notification and knowledge of hepatitis B disease [11]. However, a disparity was observed in the attitude toward prevention, with medical doctors exhibiting the least favorable attitude, followed by nurses and midwives.

Since nurses comprise the largest group of healthcare professionals directly involved in patient care [12], exploring the awareness level among nursing students regarding HBV is crucial. The objective of this study is to identify and evaluate the current levels of knowledge, attitude, and practices among nursing students in Jordanian universities concerning HBV infection.

## 2 Methodology

The study, conducted between March and August 2022, employed a cross‐sectional design among 617 nursing students. The target participants were those in the 3rd to 5th academic years who had completed an infectious diseases course at the University of Jordan and Jordan University of Science and Technology. Nursing students who are in their first or second academic years, as well as students from any other Jordanian private or public universities, were excluded. A questionnaire was formulated using Google Forms to collect responses. Participants were recruited from all nursing colleges within the specified universities using a participant‐driven sampling strategy. Ethical approval was obtained, and the questionnaire was disseminated to eligible participants through student representatives of each university. Distribution occurred through private social media groups supervised by student representatives and through direct messaging to all students in their respective academic batches. The minimum sample size required is 385, based on a 5% marginal error and a prevalence of 50% [13]. This study is part of a broader national initiative in Jordan, aiming to evaluate the levels of knowledge, attitude, and practices regarding HBV infection among healthcare students [14].

### 2.1 Questionnaire Administration

Nursing students meeting the eligibility criteria were invited to voluntarily participate by completing a questionnaire distributed via an online survey link. Electronically informed consent was obtained from all respondents, explicitly stating their right to withdraw from the survey at any point without facing any repercussions, with an assurance that no personal data would be collected.

### 2.2 Measurement Tool

The measurement tool employed was a structured English self‐administered online questionnaire based on a previously validated instrument [15]. This questionnaire comprised four sections: participant demographics, a knowledge section consisting of 43 questions, an attitude section featuring eight questions, and a practice section comprising three questions. Before dissemination, a pilot test was conducted to ensure the clarity and comprehensibility of the questions. The questionnaire is shown in File S2.

### 2.3 Ethical Considerations

The study protocol adhered to the ethical standards outlined in the Helsinki Declaration and was assessed and sanctioned by the Institutional Review Board (IRB) at the University of Jordan in Amman, Hashemite Kingdom of Jordan, on January 25, 2022 (Reference Number: 1/2022/2506 in Meeting No. 2022/1). The data were collected, processed confidentially, and stored on a personal computer accessible only to the authors.

### 2.4 Statistical Analysis

Data entry was performed using Microsoft Excel 2016 and subsequently imported into IBM SPSS Statistics (version 26.0; IBM Corp., Armonk, NY, USA) for analysis [16]. Normality of continuous variables was assessed using the Shapiro–Wilk test. Descriptive statistics were presented as means with standard deviations (SDs) for normally distributed continuous variables and as medians with interquartile ranges (IQRs) for non‐normally distributed data. Categorical variables were expressed as frequencies and percentages, with numerators and denominators reported. Associations between categorical variables, including demographic factors and KAP domains, were examined using the Chi‐square (χ²) test. To identify predictors of knowledge, attitudes, and practices (KAP) toward HBV, univariable and multivariable logistic regression analyses were performed, adjusting for potential confounding variables and incorporating significantly correlated factors. For regression analyses, adjusted odds ratios (AORs) with 95% confidence intervals (CIs) were reported to provide effect size and precision estimates. Each correct response in the questionnaire was assigned one point. Scores were categorized as good if participants answered ≥ 70% of the questions correctly in each KAP domain, and as poor if < 70% of responses were correct [14]. All statistical tests were two‐sided, and the level of significance (α = 0.05) was pre‐specified before analysis.

## 3 Results

### 3.1 Sociodemographic Factors

The demographic characteristics of the 617 participants are presented in Table 1. The mean age was 21.2 years (SD = 1.5). Of the respondents, 280 (45.4%) were male and 337 (54.6%) female. Most participants were in their 3rd year of study (352 of 617, 57.1%), followed by 4th year (178, 28.0%) and 5th year (87, 14.1%). Only 103 participants (16.7%) reported taking additional courses related to HBV. A personal history of HBV infection was reported by 6 (1.0%) students, while 19 (3.1%) had a family member with HBV. A total of 250 (40.5%) reported encountering CHB patients during their clinical practice.

**Table 1:** Demographics of survey participants (*n* = 617).

Respondent's demographics	*n*	%
Age		
Mean/SD: 21.2( ± 1.5)		
Gender		
Male	280	45.4
Female	337	54.6
Studying year		
3rd year	352	57.1
4th year	178	28
5th year	87	14.1
Extra courses about HBV		
Yes	103	16.7
No	514	83.3
History of HBV infection		
Yes	6	1
No	611	99
Family member infected with HBV		
Yes	19	3.1
No	598	96.9
Encountered CHB patient		
Yes	250	40.5
No	367	59.5

### 3.2 Knowledge About HBV

Knowledge responses are summarized in Table 2. Transmission routes were generally well recognized: 564 of 617 participants (91.4%) identified blood transfusion, and 485 (78.6%) sexual contact as routes of HBV transmission. However, misconceptions remained: only 321 (52.0%) recognized that HBV is not transmitted by coughing or sneezing, and 210 (34.0%) correctly reported that sharing food or utensils does not transmit HBV. Although two‐thirds of participants demonstrated awareness of preventing needle‐stick injuries that can transmit HBV, their responses regarding the necessity of sharp‐proof containers for proper disposal in clinics were notably inadequate (only 88 of 617, 14.3%). Knowledge about neonatal immunization was poor, with only 76 (12.3%) identifying the correct timing of the initial dose at birth. Similarly, only 50 (8.1%) identified treatment criteria for CHB. Despite this, awareness of treatment goals was higher, with 525 (85.1%) recognizing prevention of disease progression as a goal. Overall, the knowledge exhibited by students regarding HBV was assessed as satisfactory.

**Table 2:** Knowledge about HBV among health care students in Jordan (*n* = 617).

Questions	Correct answers	
	*n*	%
*Prevalence and sequelae*		
A4. What percentage of the Jordanian population has chronic hepatitis B (CHB)?	83	13.5
A5. How did most people who have CHB in Jordan get infected?	78	12.6
A6. Which age group is most likely to develop CHB after the initial infection?	85	13.8
A7. What are the consequences of chronic hepatitis B?	379	61.4
A52. Without proper monitoring and treatment, what is the chance a patient would die of CHB complications?	88	14.3
*Transmission routes*		
A8. Can hepatitis B be transmitted through handshake?	428	69.4
A9. Can hepatitis B be transmitted through unprotected sex?	485	78.6
A10. Can hepatitis B be transmitted through blood transfusion?	564	91.4
A11. Can hepatitis B be transmitted through sneezing or coughing?	321	52
A12. Can hepatitis B be transmitted through from mother to child at birth?	453	73.4
A13. Can hepatitis B be transmitted through sharing food or utensils?	210	34
*Prevention measures*		
A14. Can cleaning and cooking food thoroughly prevent HBV transmission?	211	34.2
A15. Can the hepatitis B vaccine prevent HBV transmission?	485	78.6
A16. Can HBV transmission be prevented by not reusing or sharing needles/syringes?	541	87.7
A17. Can HBV transmission be prevented by avoid sharing food/utensils or eating with a person with chronic HBV?	177	29.5
A18. Can using a condom prevent HBV transmission?	435	70.5
A19. What is the most effective preventive measure for infants born to mothers with chronic HBsAg?	126	20.4
A21. Who needs the hepatitis B vaccine?	415	67.3
A23. Prevention of mother‐to‐child transmission	132	21.4
A24. The first dose of hepatitis B vaccine for baby	76	12.3
A33. Is it necessary to have sharp‐proof containers at clinics for disposing of needles and sharp objects*?*	88	14.3
What would you do to prevent needle‐stick injury?		
A30. Wash hands with soap or disinfectant after each clinical procedure?	317	51.4
A31. Recap needle with two hands after use and discard immediately in a sharp‐proof container	425	68.9
A32. Do not recap needle and discard immediately in a sharp‐proof container	462	74.9
*Diagnosis and treatment*		
A40. What is the symptom most patients with chronic hepatitis B present with?	414	67.1
A56. Serum HBsAg test for identification of patients infected with hepatitis B virus	490	79.4
A57. What test should be used to identify immunity against the hepatitis B virus?	386	62.6
A55. When should infants born to mothers with CHB be evaluated for HBsAg status?	515	83.5
Who should be tested for hepatitis B?		
A35. Pregnant women should be tested for hepatitis B	238	38.6
A36. HIV‐infected people should be tested for hepatitis B	181	29.3
A37. Men who have sex with men (MSM) should be tested for hepatitis B	20	3.2
A38. Family members of those who have hepatitis B should be tested for hepatitis B	131	21.2
*Treatment*		
A41. What are the criteria for indicating treatment in patients with CHB?	50	8.1
A42. There is no cure, but there are effective medications to manage and control the disease?	314	50.9
What are the treatment goals for CHB patients?		
A43. Inhibit the replication of the hepatitis B virus	497	80.6
A44. Prevent disease progression of disease, particularly liver cirrhosis and liver cancer	525	85.1
A45. Prevent mother‐to‐child transmission (MTCT)	525	85.1
A46. Prevent flare of hepatitis B	515	83.5
A47. Is it true that (NAs) are a recommended first‐line treatment for CHB?	308	49.9
A48. Is treatment of CHB with NAs long term, possibly even lifetime?	311	50.4
A49. Do patients need to strictly adhere to the treatment of CHB?	429	69.5
A50. Do you think that all patients with chronic HBV need to be treated immediately?	115	18.6
A51. Should all CHB patients be monitored and tested regardless of treatment status?	372	60.3

Abbreviations: CHB, chronic hepatitis B; HBV, hepatitis B virus; MSM, men who have sex with men; MTCT, mother‐to‐child transmission; NAs, nucleotide analogs.

### 3.3 Attitude Toward HBV

Attitude responses are summarized in Table 3. Most students trusted the hepatitis B vaccine (494 of 617, 80.1%) and agreed on the necessity of vaccinating newborns at birth (385, 62.4%). Confidence in ordering laboratory tests was reported by 433 (70.2%) students, while only 197 (31.9%) felt confident prescribing treatment. Concerns about casual contact with HBV patients were relatively low: 167 (27.1%) expressed discomfort working with HBV patients, and 107 (17.3%) were concerned about sharing utensils with them.

**Table 3:** Attitude toward HBV (*n* = 617).

Questions	Answered yes	
	*n*	*%*
A20. Are you confident in counseling patients about prevention of HBV?	360	58.3
A22. Do you think that the hepatitis B vaccine is safe?	494	80.1
A25. Do you think it is necessary to vaccinate newborns for hepatitis B at birth?	385	62.4
A53. Are you confident in ordering laboratory tests to monitor CHB patients?	433	70.2
A54. Are you confident in prescribing treatment for a patient with chronic hepatitis B?	197	31.9
A58. Are you confident in ordering diagnosis tests for patients with chronic HBV?	384	62.2
A59. Would you have any concerns having casual contact or working together with a chronic HBV patient in the same office?	167	27.1
A60. Would you have any concerns sharing food or utensils with a CHB?	107	17.3

### 3.4 HBV Preventive Practices

Preventive practices are summarized in Table 4. A total of 467 of 617 participants (75.7%) reported receiving HBV vaccination before starting clinical rotations. However, only 280 (45.4%) had undergone HBV antibody testing. The consistent use of gloves during procedures was reported by 437 participants (70.8%).

**Table 4:** HBV preventive practices (*n* = 617).

Questions	Answered yes	
	*n*	%
A28. Did you get the hepatitis B vaccine before entering practicum at teaching hospitals?	467	75.7
A29. Did you get tested for HBV before entering practicum at teaching hospitals?	280	*45.4*
A34. Do you consistently wear gloves when administrating injections or performing medical procedures to patients?	437	70.8

### 3.5 Association Between Sociodemographic Factors and Knowledge, Attitude, and Practices Toward HBV

Associations between demographic characteristics and KAP outcomes are shown in Table 5. Age was significantly associated with higher knowledge, attitudes, and practices (all *p* < 0.001). Younger participants generally exhibited lower levels of knowledge and attitude but showcased better preventive practices compared with their older counterparts. Gender was associated with attitudes (*p* = 0.007) and practices (*p* = 0.001), with male students reporting higher practice scores. The academic year was associated with knowledge (*p* < 0.001), with 5th‐year students showing higher knowledge levels. Encounters with CHB patients were significantly associated with higher knowledge, attitudes, and practices (all *p* < 0.001).

**Table 5:** Association between demographic characteristics of students and knowledge, attitude, and practices toward HBV (*n* = 617).

	Level of knowledge			Level of attitude			Level of practice		
	Low (*n* = 417)	High (*n* = 200)	*p* value	Low (*n* = 330)	High (*n* = 287)	*p* value	Low (*n* = 194)	Good (*n* = 423)	*p* value
Age	21.3( ± 1.4)	22( ± 1.6)	0.000	21.4 ( ± 1.5)	21.7( ± 1.6)	0.000	21.4( ± 1.5)	21.6( ± 1.5)	0.000
Gender									
Male	195	85	0.320	133	147	0.007	69	211	0.001
Female	222	115		197	140		125	212	
Studying year									
3rd year	263	89	0.000	193	159	0.742	107	245	0.642
4th year	114	64		92	86		56	122	
5th year	40	47		45	42		31	56	
Extra courses about HBV									
Yes	60	43	0.027	48	55	0.125	168	77	0.138
No	357	157		282	232		26	346	
History of HBV infection									
Yes	6	0	0.088	1	5	0.069	1	5	0.433
No	411	200		329	282		193	418	
Family member infected with HBV									
Yes	12	7	0.675	10	9	0.940	6	13	0.990
No	405	193		320	278		188	410	
Encountered CHB patient									
Yes	145	105	0.000	112	138	0.000	57	230	0.000
No	272	95		218	149		137	193	

### 3.6 Logistic Regression Analysis

Multivariable logistic regression results are summarized in Table 6. Older age was independently associated with higher knowledge (AOR = 1.182, 95% CI: 1.023–1.366, *p* = 0.023). Male gender was significantly associated with higher knowledge (AOR = 1.449, 95% CI: 1.047–2.005, *p* = 0.025), attitudes (AOR = 1.683, 95% CI: 1.179–2.404, *p* = 0.004), and practices (AOR = 1.877, 95% CI: 1.288–2.735, *p* = 0.001). Fifth‐year students had greater knowledge compared with third‐year students (AOR = 2.041, 95% CI: 1.114–3.740, *p* = 0.021). Encounter with CHB patients was also associated with higher attitudes (AOR = 1.660, 95% CI: 1.185–2.324, *p* = 0.003) and practices (AOR = 1.877, 95% CI: 1.288–2.735, *p* = 0.001). Taking extra HBV‐related courses was associated with better knowledge (AOR = 1.635, 95% CI: 1.129–2.368, *p* = 0.011).

**Table 6:** Logistic regression analysis establishing the associations between attitude and practice among the surveyed nursing students in Jordan.

Covariates	Knowledge			Attitude			Practice		
	OR	CI	*p* value	OR	CI	*p* value	OR	CI	*p* value
Age	1.182	1.023–1366	0.023	1.060	0.952–1.181	0.278	1.027	0.912–1.157	0.658
Gender	NA								
Male				1.449	1.047–2.005	0.025	1.683	1.179–2.404	0.004
Female				Reference			Reference		
Studying year:									
3rd year	Reference			NA			NA		
4th year	1.143	0.731–1.786	0.558						
5th year	2.041	1.114–3.740	0.021						
Encountered CHB patient									
Yes	1.257	0785–2.012	0.340	1.660	1.185–2.324	0.003	1.877	1.288–2.735	0.001
No	Reference			Reference			Reference		
Extra courses about HBV				NA			NA		
Yes	1.635	1.129–2.368	2.368						
No	Reference								

*Note:* NA, not applicable, indicates that the corresponding variable was statistically insignificant in the univariate logistic regression and, therefore, was not included in the multivariate logistic regression.

## 4 Discussion

It is vital for HCWs, due to their routine exposure to patient bodily fluids, to possess adequate knowledge and positive attitudes and implement effective preventive practices to combat viral transmission. The investigation considered various factors, including academic year, participation in additional courses related to HBV, personal exposure to the virus, family history, and direct involvement in CHB patient care. Interestingly, while the nursing students demonstrated sufficient understanding of the virus's spread, diagnosis, preventive measures, and available treatments, they exhibited a deficiency in responding to questions related to the prevalence and strategies for effective HBV vaccination. Regarding their attitudes toward HBV, there was a noticeable hesitation observed in answering questions regarding interactions with HBV‐infected patients, such as sharing utensils or dining together. This hesitation contradicted their belief that thorough cleaning and cooking could prevent virus transmission. It is important to emphasize that HBV is not transmitted through casual contact such as sharing food or utensils, nor through coughing or sneezing. Similar findings were observed in separate studies conducted in Vietnam [15]. The overall trend suggested that, despite reservations about interacting with HBV‐infected patients, participants displayed a willingness to engage with them after expressing trust in vaccines and having received vaccination. The trust in vaccines significantly influenced immunization, as 75% of students had been vaccinated before starting clinical practice, impacting their potential recommendations for vaccination within the general population. In our study, participants demonstrated awareness of the vertical transmission route of hepatitis B (mother‐to‐child); however, their responses were inadequate regarding essential preventive measures and the recommended timing of the initial vaccine dose for newborns. Timely immunization, ideally within 24 h of birth, is a critical strategy for preventing HBV infection. The unsatisfactory responses in this area underscore the importance of reinforcing this preventive measure in educational and training programs. Needle‐stick injuries represent a prevalent cause of HBV among HCWs [17]. While our participants acknowledged this risk, they appeared to overlook the emphasis on safe disposal practices, potentially increasing their vulnerability to the virus. Recapping needles after use significantly contributes to accidental needle‐stick injuries [14, 18]. A considerable portion (69%) of our survey participants confessed to recapping needles before disposal, a practice discouraged in studies conducted in India due to its strong correlation with needle‐stick injuries among HCWs [18]. In a separate cross‐sectional study in Pakistan that highlighted the impact of gender and age, it was observed that male students had lower rates of accidental needle‐stick injuries compared with females [17]. This aligns with our findings, where males demonstrated better comprehension of hepatitis B than females, despite the majority of participants being female. However, our participants exhibited limited knowledge about testing men who have sex with men, whereas several other studies showcased higher efficacy rates in answering similar questions [14, 15]. The very low proportion of correct responses regarding treatment criteria and urgency of treatment is concerning. This finding suggests that additional curricular content is needed to improve students' awareness of HBV management and to prepare them for safe clinical practice.

Given that less than half of students had undergone HBV antibody testing before clinical rotations, regular serological testing is essential. Revaccination should be offered to those who do not reach protective antibody levels to ensure adequate protection among future HCWs.

As expected, 3rd‐year college students displayed less knowledge compared with 4th and 5th‐year students, indicating a unique trend in the progression of knowledge across academic years. Similar findings from a survey in Vietnam suggest that the knowledge gap between students in lower academic years versus clinical years might be due to recent courses on the subject within their curriculum. However, it remains a controversial issue, considering that students in their clinical years typically gain more exposure than their pre‐clinical peers [15]. A systematic review by Hemant A Shah et al. highlighted the benefits of patient education about hepatitis B positively impacted their attitudes toward testing, completing vaccination, and compliance with treatments. This would have only been effective if their healthcare providers were well‐versed in their knowledge and practices to properly educate and respond to patients' concerns while providing the necessary care for them. A survey conducted in Vietnam revealed deficiencies in laboratory test ordering, diagnosis, and treatment of HBV. In contrast, participants in our study demonstrated confidence in ordering appropriate tests, establishing diagnoses, and prescribing treatments for HBV patients. Nonetheless, it remains essential to emphasize epidemiological aspects such as prevalence, disease burden, prevention, immunization, testing, and treatment criteria to further enhance awareness and improve management of the virus [13]. While the majority of our study population responded positively in the survey, there's an ongoing need to improve and enhance the overall understanding of HBV and its management. The overall “Knowledge, Attitude, and Practices” (KAP) of students in Jordan regarding HBV were somewhat at the borderline, as participants displayed a notable awareness of the significant risks associated with HBV. In concordance with such results and findings, objective‐targeted curricular reformations are required with specific focus on supplementing neonatal HBV prophylaxis timing, post‐vaccination serological testing, and safe sharps disposal practices. Such enhancements, the proposed curricular focus does not only solidify the knowledge gap but also aligns with Jordan's national HBV control strategies to reduce transmission and ensure HCWs safety.

## 5 Limitations

The data collection relied on self‐reported responses and lacked direct observation. Furthermore, the responses were obtained from only two settings from about 32 universities in Jordan with undergraduate and post‐graduate nursing programs, making it challenging to generalize the findings to a broader population.

Participant‐driven sampling makes our paper prone to selection bias. The choice of such sampling was due to the lack of sufficient epidemiological data in the kingdom towards the extent of HBV understanding among the nursing students.

Future research endeavors are encouraged to encompass a more comprehensive representation by including both public and private sectors, further enhancing the understanding of HBV‐related KAP among various segments of the population.

## 6 Conclusion

HCWs, particularly nurses, maintain frequent interaction with HBV patients. Enhancing the understanding of knowledge, attitude, and practices toward ailments, such as HBV, is pivotal. This study highlighted a strong correlation between Knowledge, Attitude, and Practices (KAP) among the participants, signifying ample room for advancement across all three dimensions. The foundation of exposure to HBV often begins in medical or nursing schools, primarily through the imparting of knowledge, which forms the basis of their attitude toward the subject and significantly influences their future practices.

### Implications for Health Policies

With all the provided results in this article, we put forward the recommendation to integrate more comprehensive educational materials focusing on hepatitis B into nursing training programs. By fortifying educational curricula, we can better equip future healthcare professionals in effectively managing and preventing the spread of HBV.

## Author Contributions

Conceptulaizaition: N.A. and A.H.A. Methodology: N.A., A.H.A, R.M.J, R.F.J., A.Y.M. and H.N. Data Curation and formal analysis: R.M.J. R.F.J., R.A.J. and M.A. Investigation: N.A., H.N., M.A. and M.J. writing – original draft preparation: R.M.J., R.F.J., A.B., M.A., H.N., and M.J. Writing – review and editing, N.A., R.A.J., R.M.J., R.F.J., A.B., M.A., H.N., M.J., N.A.H., M.N.A., H.A., L.I., A.Y.M., H.M. and A.H.A. Supervision: N.A and A.H.A. Project adminstration: N.A., A.H.A., R.M.J. and R.F.J. All authors have reviewed and agreed to the published version of the manuscript.

## Funding

The authors received no specific funding for this work.

## Ethics Statement

The protocol and consent form of the study were developed in accordance with the Helsinki Declaration's ethical standards, and it had been approved and reviewed by the Institutional Review Board (IRB) at the University of Jordan on (1/25/2022) (reference number: 1/2022/2506). All respondents were given written informed consent before completing the questionnaire.

## Consent

Informed consent was obtained from participants to participate in the study.

## Conflicts of Interest

The authors declare no conflicts of interest.

## Transparency Statement

The lead author, Nader Alaridah, affirms that this manuscript is an honest, accurate, and transparent account of the study being reported; that no important aspects of the study have been omitted; and that any discrepancies from the study as planned (and, if relevant, registered) have been explained.

## Supporting information

Supporting File 1 (Respondents). The file displays the respondents' numbers and percentages for each given question in the questionnaire.

Supporting File 2 (Survey). The file contains the English and Arabic versions of the given questionnaire. All authors have read and approved the final version of the manuscript. Nader Alaridah had full access to all of the data in this study and takes complete responsibility for the integrity of the data and the accuracy of the data analysis. Nader Alaridah affirms that this manuscript is an honest, accurate, and transparent account of the study being reported; that no important aspects of the study have been omitted; and that any discrepancies from the study as planned (and, if relevant, registered) have been explained.

## Data Availability

The data that support the findings of this study are available in the Supporting Material of this article.
