# Supplementary material for: Knowledge, Attitude, and Practices Towards Hepatitis B Infection Among Nursing Students: A Cross‐Sectional Study in Jordan
Source: Health Sci Rep. 2026 Mar 4;9(3):e71967. doi: 10.1002/hsr2.71967 (PMC12959464; doi:10.1002/hsr2.71967)
Supplement: Supplementary file 1 — Supporting File 1 (Respondents). The file displays the respondents' numbers and percentages for each given question in the questionnaire. [file HSR2-9-e71967-s002.docx]

Table 1. Knowledge about HBV among health care students in Jordan (n = 617).

| Questions | Correct answers | |
| --- | --- | --- |
|  | n | % |
| Prevalence and Sequelae | | |
| A4. What percentage of the Jordanian population has chronic hepatitis B (CHB)? | 83 | 13.5% |
| A5. How did most people who have CHB in Jordan get infected? | 78 | 12.6% |
| A6. Which age group is most likely to develop CHB after the initial infection? | 85 | 13.8% |
| A7.What are the consequences of chronic hepatitis B? | 379 | 61.4% |
| A52. Without proper monitoring and treatment, what is the chance a patient would die of CHB complications? | 88 | 14.3% |
| Transmission Routes | | |
| A8. Can hepatitis B be transmitted through handshake? | 428 | 69.4% |
| A9. Can hepatitis B be transmitted through unprotected sex? | 485 | 78.6% |
| A10. Can hepatitis B be transmitted through blood transfusion? | 564 | 91.4% |
| A11. Can hepatitis B be transmitted through sneezing or coughing? | 321 | 52% |
| A12. Can hepatitis B be transmitted through from mother to child at birth? | 453 | 73.4% |
| A13. Can hepatitis B be transmitted through sharing food or utensils? | 210 | 34% |
| Prevention Measures | | |
| A14. Can cleaning and cooking food thoroughly prevent HBV transmission? | 211 | 34.2% |
| A15. Can the hepatitis B vaccine prevent HBV transmission? | 485 | 78.6% |
| A16. Can HBV transmission be prevented by not reusing or sharing needles/syringes? | 541 | 87.7% |
| A17. Can HBV transmission be prevented by avoid sharing food/utensils or eating with a person with chronic HBV? | 177 | 29.5% |
| A18. Can using a condom prevent HBV transmission? | 435 | 70.5% |
| A19.What is the most effective preventive measure for infants born to mothers with chronic HBsAg? | 126 | 20.4% |
| A21. Who needs the hepatitis B vaccine? | 415 | 67.3% |
| A23. Prevention of mother-to-child transmission | 132 | 21.4% |
| A24. The first dose of hepatitis B vaccine for baby | 76 | 12.3% |
| A33. Is it necessary to have sharp-proof containers at clinics for disposing of needles and sharp objects? | 88 | 14.3% |
| What would you do to prevent needle-stick injury? | | |
| A30. Wash hands with soap or disinfectant after each clinical procedure? | 317 | 51.4% |
| A31. Recap needle with two hands after use and discard immediately in a sharp-proof container | 425 | 68.9% |
| A32. Do not recap needle and discard immediately in a sharp-proof container | 462 | 74.9% |
| Diagnosis and Treatment | | |
| A40. What is the symptom most patients with chronic hepatitis B present with? | 414 | 67.1% |
| A56. Serum HBsAg test for identification of patients infected with hepatitis B virus | 490 | 79.4% |
| A57. What test should be used to identify immunity against the hepatitis B virus? | 386 | 62.6% |
| A55. When should infants born to mothers with CHB be evaluated for HBsAg status? | 515 | 83.5% |
| Who should be tested for hepatitis B? | | |
| A35. Pregnant women should be tested for hepatitis B | 238 | 38.6% |
| A36. HIV-infected people should be tested for hepatitis B | 181 | 29.3% |
| A37. Men who have sex with men (MSM) should be tested for hepatitis B | 20 | 3.2% |
| A38. Family members of those who have hepatitis B should be tested for hepatitis B | 131 | 21.2% |
| Treatment | | |
| A41. What are the criteria for indicating treatment in patients with CHB? | 50 | 8.1% |
| A42. There is no cure, but there are effective medications to manage and control the disease? | 314 | 50.9% |
| What are the treatment goals for CHB patients? |  |  |
| A43. Inhibit the replication of the hepatitis B virus | 497 | 80.6% |
| A44. Prevent disease progression of disease, particularly liver cirrhosis and liver cancer | 525 | 85.1% |
| A45. Prevent mother-to-child transmission (MTCT) | 525 | 85.1% |
| A46. Prevent flare of hepatitis B | 515 | 83.5% |
| A47. Is it true that (NAs) are a recommended first-line treatment for CHB? | 308 | 49.9% |
| A48. Is treatment of CHB with NAs long term, possibly even lifetime? | 311 | 50.4% |
| A49. Do patients need to strictly adhere to the treatment of CHB? | 429 | 69.5% |
| A50. Do you think that all patients with chronic HBV need to be treated immediately? | 115 | 18.6% |
| A51. Should all CHB patients be monitored and tested regardless of treatment status? | 372 | 60.3% |

Abbreviations: CHB: chronic hepatitis B; HBV: hepatitis B virus; MSM: men who have sex with men; MTCT: mother-to-child transmission; NAs: Nucleotide Analogs.

Table 2. Attitude toward HBV (n = 617).

| Questions | Answered yes | |
| --- | --- | --- |
|  | n | % |
| A20. Are you confident in counseling patients about prevention of HBV? | 360 | 58.3% |
| A22. Do you think that the hepatitis B vaccine is safe? | 494 | 80.1% |
| A25. Do you think it is necessary to vaccinate newborns for hepatitis B at birth? | 385 | 62.4% |
| A53. Are you confident in ordering laboratory tests to monitor CHB patients? | 433 | 70.2% |
| A54. Are you confident in prescribing treatment for a patient with chronic hepatitis B? | 197 | 31.9% |
| A58. Are you confident in ordering diagnosis tests for patients with chronic HBV? | 384 | 62.2% |
| A59. Would you have any concerns having casual contact or working together with a chronic HBV patient in the same office? | 167 | 27.1% |
| A60. Would you have any concerns sharing food or utensils with a CHB? | 107 | 17.3% |

Table 3. HBV Preventive Practices (n = 617).

| Questions | Answered yes | |
| --- | --- | --- |
|  | n | % |
| A28. Did you get the hepatitis B vaccine before entering practicum at teaching hospitals? | 467 | 75.7% |
| A29. Did you get tested for HBV before entering practicum at teaching hospitals? | 280 | 45.4% |
| A34. Do you consistently wear gloves when administrating injections or performing medical procedures to patients? | 437 | 70.8% |
